# Effect of an In-Home Palivizumab Administration Program for Children with Medical Complexity

**DOI:** 10.3390/children11101171

**Published:** 2024-09-26

**Authors:** Hina Emanuel, Aravind Yadav, Julie C. Eapan, Maria Caldas-Vasquez, Tomika S. Harris, Katrina McBeth, Fatima Boricha, Janice John, Ivan G. Magana Ceballos., Giuseppe N. Colasurdo, Maria E. Tellez, Tina Reddy, Wilfredo De Jesús-Rojas, Ricardo A. Mosquera

**Affiliations:** 1 Department of Pediatrics, Division of Pulmonary Medicine, McGovern Medical School, University of Texas Health Science Center, Houston, TX 77030, USA; hemanuel@stanford.edu (H.E.); aravind.yadav@uth.tmc.edu (A.Y.); julie.eapan@uth.tmc.edu (J.C.E.); maria.c.caldasvasquez@uth.tmc.edu (M.C.-V.); tomika.s.harris@uth.tmc.edu (T.S.H.); katrina.e.mcbeth@uth.tmc.edu (K.M.); fatima.m.boricha@uth.tmc.edu (F.B.); janice.l.john@uth.tmc.edu (J.J.); ivan.g.maganaceballos@uth.tmc.edu (I.G.M.C.); guiseppe.n.colasurdo@uth.tmc.edu (G.N.C.); marie.e.tellez@uth.tmc.edu (M.E.T.); tina.reddy@uth.tmc.edu (T.R.); 2 Department of Pediatrics and Basic Sciences, Ponce Health Sciences University, Ponce, PR 00716, USA; wilfredo.dejesus3@upr.edu

**Keywords:** children with medical complexity, respiratory syncytial virus, palivizumab

## Abstract

Background: In-home palivizumab administration programs (PH) have shown promise in reducing RSV-associated infections. These programs may be particularly beneficial for children with medical complexity (CMC) by limiting their exposure to healthcare-associated infections (HAIs) from non-RSV-related pathogens during transportation and visits to medical facilities. Methods: In this prospective study, 41 children with CMC less than 2 years of age were randomized by their health insurance to receive PH or in the clinic (PC) during the RSV season (October 2018–April 2019). Patients were stratified by home ventilation. The primary outcome was the total number of face-to-face encounters. Secondary outcomes were unscheduled clinic visits and hospitalizations secondary to the non-RSV LRTIs. Standard frequentist and Bayesian analyses were performed. Results: All demographic factors and strata were matched between PH (“*n*” = 13, mean age 22 mo. SD ± 1), and PC (“*n*” = 28, mean age: 18 mo. SD ± 1). There was a decrease in the number of total face-to-face encounters (adjusted for mechanical ventilation and baseline diagnosis) [(4.5 vs. 8.8), (RR: 1.8, 95% CI: 1.3–2.5, *p* = 0.001)], and hospitalizations [(0.3 vs. 1.25), (RR: 3.8, 95% CI: 1.3–11.3, *p* = 0.016)], in the PH vs PC groups. Bayesian analysis showed a 93% probability of benefit in favor of fewer face-to-face encounters in the PH group. Conclusions: This study suggests that PH administration may reduce healthcare utilization in CMC. Minimizing exposure to healthcare facilities and supporting home-based interventions are promising strategies for this population.

## 1. Introduction

Children with medical complexity (CMC) represent less than 1% of all children in the U.S. [1]. There is an increasing trend towards healthcare utilization in CMC, especially in the first five years of establishing a medical home [2,3]. Multiple subspecialist visits and inpatient admissions increase the risk of healthcare-acquired infections (HCAIs) [4,5]. 

Respiratory syncytial virus (RSV) is one of the leading causes of lower respiratory tract infections (LRTIs) in infants and young children [6]. Nosocomial RSV-related morbidity and mortality are highest in children with underlying medical illnesses [7,8,9]. CMC are susceptible to respiratory illness and RSV-related hospitalization due to multisystem disorders, compromised airways, and weakened immune systems [10,11].

Palivizumab is recommended to at-risk children during RSV season by the American Academy of Pediatrics (AAP) [12]. CMC visits to healthcare facilities for palivizumab administration increase their exposure to medical settings. They require a medical transportation vehicle, travel medical equipment, and a home health nurse or guardian, resulting in financial and social constraints. Home-based programs for CMC have been shown to decrease ER visits, length of hospital stay, and hospitalizations, focusing on mitigating families’ challenges in navigating complex healthcare systems and collaboration with inpatient and subspecialty providers [13,14]. Despite this, CMC have disproportionally high hospital use due to improved survival [3,15,16].

Administration in the home setting has been shown to decrease RSV-associated hospitalization rates in the 2000 to 2004 Palivizumab Outcomes Registry [17]. At-home prophylaxis of palivizumab showed favorable outcomes in reducing RSV-related infections, lowering rates of unscheduled medical visits and all-cause hospitalizations, increasing parent satisfaction, and reducing healthcare resource utilization [18,19,20]. There is limited literature to support the impact of home-based programs on overall healthcare utilization in CMC. However, it has been observed in other situations that telemedicine in comprehensive care provided by CMC at patients’ homes offers significant benefits. These include a reduction in days of treatment outside the hospital environment, fewer severe illnesses, decreased incidence of non-RSV-related diseases, and lower health system costs while significantly increasing access, direct contact, and follow-up with the primary care provider [21]. At the time of this writing, the standard practice was to administer palivizumab to CMC at the clinic.

We hypothesized that at-home monthly palivizumab administration would decrease the need for healthcare utilization in CMC. This would be attributed to a lower risk of acquiring non-RSV-related infections during transportation and visits to healthcare facilities. The primary outcome was a total number of face-to-face encounters (scheduled and unscheduled patient-to-physician contact) in patients administered palivizumab at home (PH) and in the clinic (PC). The secondary outcomes included the total number of clinic visits, unscheduled clinic visits, hospitalizations (including intensive care unit admissions), emergency room visits, hospital days, and hospitalizations due to non-RSV LRTIs, all analyzed on a per-patient basis.

## 2. Materials and Methods

In this prospective study, 41 CMC less than 2 years of age seen at The University of Texas High-Risk Children’s Clinic between October 2018 and April 2019 (RSV season) were enrolled after approval by the Institutional Review Board (HMS-19-0565) as a quality improvement project. This study was conducted in strict adherence to the principles of the Declaration of Helsinki. Data collection involved an analysis of patient medical records supplemented by direct communication with parents or caregivers. All patient information was handled with the utmost confidentiality, ensuring anonymity throughout the research process. All participants qualified for RSV prophylaxis under AAP guidelines. Consent was obtained from the parents to administer palivizumab at home. Approval was obtained from the respective health insurance companies for palivizumab administration at home. This approach did not incur any additional financial burden on our clinic, as healthcare payers fully covered all costs. This included the provision of a nurse to administer palivizumab during monthly home visits throughout the winter or RSV season.

CMC were defined as patients with chronic illnesses requiring at least two medical subspecialists with more than 1 intensive care unit admission, more than 2 hospital admissions regardless of intensive care unit admission, and/or 3 emergency department visits in one year with anticipated admissions in the next year. CMC eligible for palivizumab administration were children with chronic lung disease or prematurity, neuromuscular, and hemodynamically significant cardiac disease. Data on the palivizumab administration was obtained from home health nurses and insurance companies for CMC in the PH group and from direct physician-to-patient contact in the PC group. The total number of face-to-face encounters, including total clinic visits, unscheduled clinic visits, hospitalizations including intensive care unit admissions, ER visits, hospital days, and hospitalizations secondary to the non-RSV LRTIs to complete the primary and secondary outcomes, were collected from the electronic medical record by assessing the Children’s Memorial Hermann Hospital and data from the 14 hospitals in the Memorial Hermann Hospital System. In addition, we asked the parents, primary caregivers, and health nurses if, during the length of the study, they, for any reason, went to any other clinic or health facility besides the University of Texas High-Risk Children Clinic.

Individuals were quasi-randomized by health insurance payers after submitting the request for home palivizumab administration for all participating patients into two groups: (1) the PH group and (2) the PC group. Participants were categorized into PH or PC groups if at least 3 doses of palivizumab were administered in the home vs. clinic, respectively. Individuals in both groups were further stratified into positive pressure (invasive or noninvasive ventilatory support) and without positive pressure. The sample size calculation was based on an anticipated 50% difference in total medical visits outside the home (a composite outcome including ER visits, clinic visits, and hospital days) between the in-home palivizumab administration group and the in-clinic administration group. To detect this 50% difference in the mean of the primary outcome with 80% power at a two-sided significance level of 0.05, a sample size of approximately 32 patients per group was determined to be necessary; the actual enrollment resulted in 13 patients in the in-home palivizumab administration group and 28 in the in-clinic administration group. Baseline diagnosis was matched between PH and PC groups, with one participant in PH to two participants in the PC group.

Data analysis: Standard frequentist and Bayesian analyses were performed using an intent-to-treat approach analysis. The total number of days of unscheduled face-to-face encounters (counting one day for each visit), total clinic visits (counting one day for each visit), total unscheduled clinic visits (counting one day for each visit), total hospital admissions (counting each hospitalization as an event), total ER visits (counting one day for each ER visit), total hospital days, and hospitalization due to non-RSV LRTIs were analyzed in both groups.

Frequencies (with percentages) were used to describe the categorical variables. Descriptive statistic of the mean was used for continuous variables. All statistical testing was performed in Stata/IC v.16.0 (Stata Corp, College Station, TX, USA), and statistical significance was assumed at a two-sided alpha of 0.05.

Negative binomial regression models with robust standard errors (SEs) to estimate relative risks (RRs) and 95% confidence intervals (CIs) were performed. We used Bayesian models between groups (PH and PC) to assess the probability of benefit. Bayesian analysis was performed for the primary outcome. For Bayesian analyses, neutral prior distributions for all regression coefficients were centered at a relative risk (RR) of 1.0 (normal with a mean of 0 and SD of 1 on the log scale) and half-normal (0, 1) for SD parameters. Point estimates of treatment effect and 95% credible intervals and the probability of treatment benefit were reported.

## 3. Results

Of 41 enrolled patients, 13 (32%) were in the PH group, and 28 (68%) were in the PC group. The two groups did not differ in demographics and baseline characteristics. After adjusting for baseline diagnosis and mechanical ventilation, the groups were analyzed for healthcare utilization (Table 1).

Overall, the common admitting problems in all participants were respiratory 41% (*n* = 17/41), gastrointestinal 3% (*n* = 1/41), and gastrointestinal and respiratory combined 17% (*n* = 7/41). None of the patients in both groups were admitted secondary to RSV. However, non-RSV LRTI hospitalizations were seen in both groups with increasing frequency in the PC group (PH 4 vs. PC 13, OR: 1.6, 95% CI: 0.5–4.7, *p* = 0.379). The PH group had a significant decrease in the average number of face-to-face encounters (PH 4.5 vs. PC 8.8, RR: 1.8, 95% CI: 1.3–2.5, *p* = 0.001) compared to the PC group. A similar trend in a significant decrease in the average number of total clinic visits (PH 4.1 vs. PC 7, RR: 1.6, 95% CI: 1.2–2.2, *p* = 0.004) including unscheduled clinic visits (PH 1.2 vs. PC 1.8 RR: 2, 95% CI: 0.9–4.1, *p* = 0.051) and ER visits (PH 0.4 vs. PC 1.8, RR: 3.8, 95% CI: 1.5–9.6, *p* = 0.004) was reported in the PH group. Furthermore, the average number of hospitalizations was significantly reduced (PH 0.3 vs. PC 1.2, RR: 3.8, 95% CI: 1.3–11.3, *p* = 0.016) with shorter hospital stays in the PH group (PH 2 vs. PC 12 days RR: 6.5, 95% CI: 0.9–46.5; *p* = 0.064) (Table 2). The Bayesian analysis showed a 93% probability of benefit in favor of fewer total face-to-face encounters in the PH group.

## 4. Discussion

The in-home-based palivizumab program resulted in a decrease in healthcare utilization, including scheduled and unscheduled clinic visits, ER visits, and all-cause hospitalization in the cohort of CMC. These results remained consistent after adjusting for baseline diagnosis and the need for mechanical ventilation (positive pressure vs no positive pressure). Our finding highlights the unique model that emphasizes the importance of providing care at home for CMC, reducing infectious exposure to the healthcare delivery system.

Medical complexity in these children necessitates frequent access to healthcare services, increasing the risk of HCAIs. Susceptibility for acquiring HCAIs in CMC includes prior colonization with healthcare-acquired microorganisms, prior antibiotic use with the eradication of normal flora, and contamination of inhalation and feeding equipment [22]. RSV has been shown as a significant nosocomial pathogen in pediatric hospital units [23]. Palivizumab administration in CMC effectively decreased RSV infections in our patient population. However, CMC were admitted due to non-RSV LRTIs, gastrointestinal, combined gastrointestinal, and respiratory conditions. We speculate the role of HCAIs as a contributing factor towards increased healthcare utilization in CMC. The integrative review of telemedicine in CMC supports the favorable outcomes of reduced unplanned hospitalizations, readmissions, length of hospital stay, and unplanned clinic visits [24]. It is possible that this finding could be reflective of reduced exposure of patients to the healthcare facility and, therefore, decreased risk of HCAIs. 

The home-based palivizumab program has been implemented in the Netherlands and Ireland. Still, due to system-wide quality improvement projects, there were no control groups to assess the efficacy of home-based vaccine programs in at-risk children. Our study participants were quasi-randomized, making selection bias and threats to internal validity a possibility; however, the participants in each group were similar in terms of their baseline characteristics. The home-based palivizumab administration incurred no additional cost. We used a single healthcare electronic medical record for outcomes. As all participants were closely followed at our clinic, the potential of missing information (ER visits, hospitalizations, and clinic visits) was minimized. Our study evaluated outcomes for only one RSV season; it is unclear if results would have been sustained over additional seasons. Although results were limited to one RSV season, enrollment time in the study was the same for both groups, eliminating the difference between peak RSV acquisition and antibody titers. This study did not conduct surveys to evaluate parental satisfaction and quality of life. Another limitation of our study was the relatively small sample size.

## 5. Conclusions

In conclusion, the study demonstrates that an in-home palivizumab program significantly reduces healthcare utilization in children with medical complexity by decreasing scheduled and unscheduled clinic visits, emergency room visits, and all-cause hospitalizations. This reduction is observed even after adjusting for baseline health conditions and the necessity for mechanical ventilation. The findings highlight the effectiveness of home-based care in minimizing exposure to infectious agents within healthcare settings, thereby reducing the risk of healthcare-associated infections. This study also wants to emphasize the critical need to minimize the exposure of CMC to environments with a high risk of healthcare-associated infections, with the importance of implementing targeted educational interventions for families and administering age-appropriate vaccination schedules within the home setting, which are essential for CMC patients. These measures could significantly reduce pathogen exposure, thereby decreasing the incidence of infections and the risk of complications associated with the multiple comorbidities that characterize this vulnerable population. While the smaller sample size may limit the study’s power to detect statistically significant differences, it does not invalidate the findings. This study can still provide meaningful preliminary data and help identify trends that could be further explored in future research with a larger sample size. Further research could expand on these findings by exploring long-term outcomes over multiple RSV seasons and assessing parental satisfaction and quality of life impacts.

## Figures and Tables

**Table 1:** Demographics and Baseline Diagnosis.

Characteristics	PH Group (*n* = 13)	PC Group (*n* = 28)	Total (*n* = 41)	*p* Value
Age in months, meant (SD)	22 (±1)	18 (±1)	20 (±1)	0.264
Male, *n* (%)	9 (22)	20 (49)	29 (68)	0.123
Female, *n* (%)	4(10)	8 (19)	12 (29)	0.325
Ethnicity				
Hispanic, *n* (%)	6 (15)	21 (51)	27 (66)	0.191
Caucasian, *n* (%)	3 (7)	5 (12)	8 (19)	0.418
Other, *n* (%)	2 (5)	4 (10)	6 (15)	0.523
Baseline Diagnosis				
Chronic lung disease, *n* (%)	7 (17)	16 (39)	23 (56)	0.175
Neuromuscular disease, *n* (%)	4 (10)	8 (19)	12 (29)	0.325
Cardiac disease, *n* (%)	2(5)	4 (10)	6 (15)	0.523
Positive pressure, *n* (%)	4 (10)	6 (15)	10 (24)	0.853

**Table 2:** Healthcare Utilization among CMC.

Characteristics	PH Group (*n* = 13)	PC Group (*n* = 28)	RR (95% CI)	*p* Value
Total face-to-face encounters, mean	4.5	8.8	1.8 (1.3–2.5)	0.001
Total clinic visits, mean	4.1	7	1.6 (1.2–2.2)	0.004
Unscheduled clinic visits, mean	1.2	1.8	2 (0.9–4.1)	0.051
ER * visits, mean	0.4	1.8	3.8 (1.5–9.6)	0.004
Hospitalizations, mean	0.3	1.2	3.8 (1.3–11.3)	0.016
Hospital days, mean	2	12	6.5 (0.9–46.5)	0.064

* ER = Emergency room.

## Data Availability

The raw data supporting the conclusions of this article will be made available by the authors on request.

## Abbreviations

PH	In-home palivizumab administration
CMC	Children with medical complexity
HAIs	healthcare-associated infections
PC	In-clinic palivizumab administration
RSV	Respiratory syncytial virus
LRTIs	Low respiratory tract infections
AAP	American Academy of Pediatrics
